# Monitoring and management of CMV and EBV after autologous haematopoietic stem cell transplantation for autoimmune diseases: a survey of the EBMT Autoimmune Diseases Working party (ADWP)

**DOI:** 10.1038/s41409-024-02461-6

**Published:** 2024-11-07

**Authors:** Tobias Alexander, Manuela Badoglio, Myriam Labopin, Thomas Daikeler, Dominique Farge, Majid Kazmi, Montserrat Rovira, Elisa Roldan, John Snowden, Greco Raffaella, On behalf of the Autoimmune Diseases Working Party (ADWP) of the EBMT, Tobias Alexander, Manuela Badoglio, Myriam Labopin, Thomas Daikeler, Dominique Farge, Majid Kazmi, Montserrat Rovira, Elisa Roldan, John Snowden, Greco Raffaella

**Affiliations:** 1 Department of Rheumatology and Clinical Immunology, Charité - Universitätsmedizin Berlin, Berlin, Germany; 2 EBMT Paris study office, Department of Hematology, Saint Antoine Hospital, INSERM UMR 938, Sorbonne University, Paris, France; 3 Department of Rheumatology University Hospital Basel, Basel, Switzerland; 4 AP–HP, hôpital St-Louis, centre de référence des maladies auto-immunes systémiques rares d’Île-de-France MATHEC (FAI2R), Unité de Médecine Interne (UF 04): CRMR MATHEC, Maladies auto-immunes et thérapie cellulaire (UF 04), Paris, France; 5 Université de Paris, IRSL, Recherche clinique appliquée à l’hématologie, URP-3518, Paris, France; 6 Department of Haematology, Guy’s and St. Thomas’ NHS Foundation Trust, London, UK; 7 Department of Haematology, Hospital Clinic, Barcelona, Spain; 8 Department of Haematology, Sheffield Teaching Hospitals NHS Foundation Trust & University of Sheffield, Sheffield, UK; 9 Unit of Hematology and Bone Marrow Transplantation, IRCCS San Raffaele Hospital, Vita-Salute San Raffaele University, Milan, Italy

**Keywords:** Health care, Immunology

**TO THE EDITOR:**

Cytomegalovirus (CMV) and Epstein-Barr virus (EBV) infections and diseases are infrequent but considerable complications among autologous haematopoietic stem cell transplantation (HCT) recipients [1, 2]. Guidelines have been developed for the disease monitoring and management in haematologic indications [3, 4], and have also featured in specific guidelines for autoimmune diseases [5]. However, adherence to recommendations may vary, as shown by the results of a recent survey conducted by the Infectious Diseases Working Party (IDWP) of the European Society for Blood and Marrow Transplantation (EBMT) on the management of CMV infection after allogeneic HCT [6]. Given the increasing activity in autologous HCT for autoimmune diseases (ADs) [7], there is a need to understand transplant centre policy for monitoring and management of CMV and EBV reactivations. In this context, the use of serotherapy as part of the conditioning regimen, commonly anti-thymocyte globulin, may represent a risk factor for viral infections after autologous HCT [8]. We therefore undertook a survey to investigate the real world of EBMT practice in centres performing autologous HCT for ADs.

A 30-item questionnaire covering aspects of diagnosis, monitoring, prophylaxis and therapy for CMV and EBV, previously approved by the scientific board of Autoimmune Diseases Working Party (ADWP), was sent to 125 centres performing auto-HCT for ADs across EBMT between 1994 and 2022. A reply to the questionnaire was received from 55 of the 125 centres (44%). The responding centres were from all over the world and included 19 countries (Table S1). 46 centres (84%) had JACIE accreditation. 41 centres (75%) performed HCT for adults only, 2 (4%) for paediatric and 12 (22%) for both. Overall, these centres performed 3286 transplants for AD between 1994 and 2022, together covering about 71% of transplants reported to the EBMT registry during this period. 56% of the centres used the same protocol for monitoring CMV or EBV in autologous HCT for AD and haematological indications, with the vast majority (>82%) of remaining centres monitoring HCT recipients only in AD patients. Main results of the survey are summarized in Fig. 1.

**Fig. 1:**
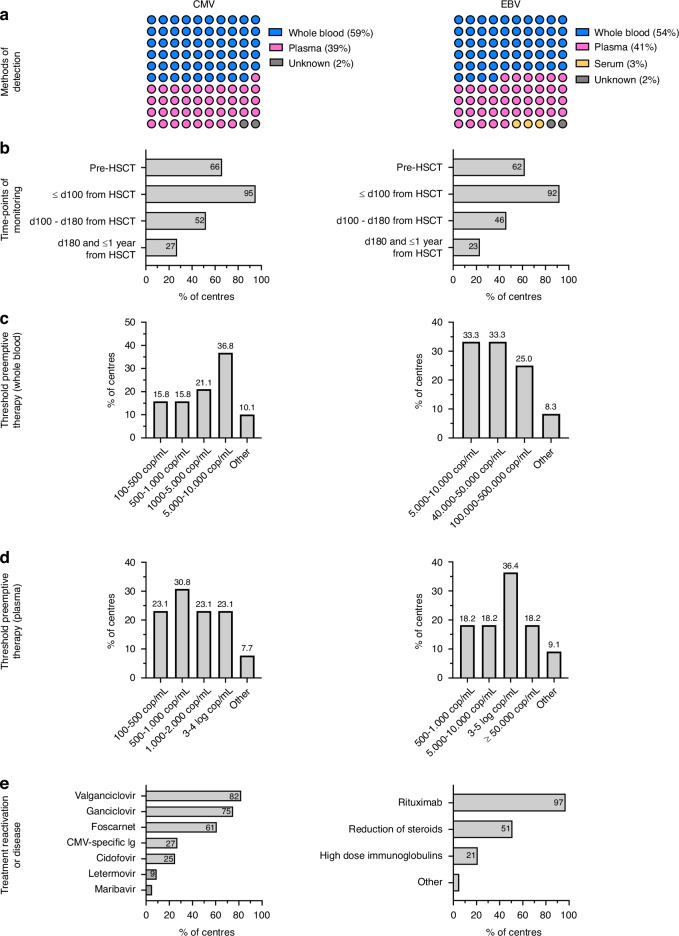
Details on centre policy for monitoring and treatment of CMV and EBV reactivations resulting from the survey. **a** Methods of detecting CMV or EBV vireamia, **b** time point of monitoring CMV or EBV, **c** threshold for pre-emptive therapy (detection in plasma), **d** threshold for pre-emptive therapy (detection in whole blood), **e** treatment for reactivation of CMV or EBV.

## CMV surveillance, prophylaxis and treatment

Serology testing prior to autologous HCT was routinely performed by 91% of centers. 45 of 51 centres (82%) performed CMV surveillance in all HCT recipients for AD, from which 98% used PCR for CMV DNA monitoring, 26 (59%) of centres using whole blood and 17 (39%) using plasma as source, respectively. CMV monitoring was performed by 42 centres (95%) until d100, by 23 (52%) until d180 and 12 (27%) until 1-year post auto-HCT. During the first 100 days, the frequency of surveillance was once a week in 14 (31%), twice in case of high-risk patients, i.e., patients with CMV DNA positivity, by 12 (25%), and weekly only in the first month followed by every other week by 14 centres (31%). Drug prophylaxis was performed by all centres, using acyclovir in 28 (64%), valganciclovir in 15 (34%) and ganciclovir in 1 centre (2%). A cut-off value for starting pre-emptive therapy was established by 31 centres (70%), but with varying threshold values (Fig. 1c, d, left panel). The choice of drugs used for CMV reactivation or disease (multiple answers allowed) included valganciclovir in 36 (82%), ganciclovir in 33 (75%), foscarnet in 27 (61%), CMV-specific immunoglobulins in 12 (27%), cidofovir in 11 (25%), letermovir in 4 (9%) and maribavir in 2 (5%) of centres.

## EBV surveillance prophylaxis and treatment

Monitoring of EBV viremia in auto-HCT recipients was reported by 39/51 (76%) of centres and all of them tested EBV-specific serology before HCT. 38 of 39 centres (97%) used PCR for EBV DNA monitoring, with 21 (55%) of centres using whole blood, 16 (41%) using plasma, and 1 (3%) serum as source, respectively. Until d100, EBV monitoring was performed by 36 (92%), until d180 by 18 (46%) and until 1 year by 9 centres (32%). During the first 100 d, PCR was performed weekly by 15 (39%) every other week by 5 (13%), once weekly for the first months followed by every other week in 12 (31%), and twice a week in high-risk patients by 4 centres (10%). A cut-off value for starting pre-emptive therapy was established by 25 (64%) of centres. The most frequently used value for initiating pre-emptive therapy was a threshold of >3–5 log copies/mL, which, however, varied greatly from centre to centre (Fig. 1c, d, right panel). Rituximab was the first line treatment both for pre-emptive and EBV disease therapy applied by 38 (97%), along with reduction in steroids performed by 20 (51%) and administration of immunoglobulins used by 8 centres (21%).

The use of antiviral drugs for CMV antigenemia or viremia represented a milestone in the past years to reduce the incidence of CMV end-organ disease after HCT for haematologic malignancies. The advent of letermovir (LTV), a CMV-specific antiviral terminal complex recently approved for CMV prophylaxis during allogeneic HSCT [9], determined a significant decrease of CMV-reactivation. For EBV no anti-replicating drug is available and commonly B cell targeting antibodies such as rituximab are used to deplete EBV-transformed B cells that lead to post-transplant lymphoproliferative disease (PTLD) or are main reservoirs for EBV. In this context, early administration of rituximab has been demonstrated to decreased the incidence and mortality of PTLD [10]. In order to ensure timely initiation of treatment in the event of viral reactivation, continuous and precise monitoring of the viral load is required. In this context, harmonized monitoring for CMV reactivation has been shown to improve risk stratification and management of recurrent cytomegalovirus reactivation after allogeneic HCT [11]. These data underscore the importance of standard operating procedures in all centers for the monitoring and treatment of viral reactivations following HCT.

This survey, conducted in 2023 amongst EBMT centres, reveals a high degree of consistency regarding the monitoring and management policies implemented for CMV and EBV infection and/or diseases in autologous HCT recipients for ADs. Most participating centres reported routine PCR-based surveillance for CMV (82% of centres) and EBV (76% of centres) viremia, with the vast majority (>92%) covering the first 100 d post-transplant. The greatest differences between the centers were found in the frequency and PCR methods for monitoring and the cut-off values for the initiation of pre-emptive therapy. Most centres perform weekly monitoring, but with variability according to risk factors. Whole blood was used as the type of sample for monitoring by only 59% of centres for CMV and 54% for EBV, respectively, although being reported to be more sensitive for detection compared to plasma [12]. The preferred threshold for CMV was a load of >5000 copies/mL in whole blood and >500 copies/mL in plasma. This differs somewhat from the survey results of the EBMT IDWP, in which the most-frequent cut-off was a CMV load >10^3^ copies/mL or >10^3^ IU/mL, probably related to the allogeneic setting [6].

In conclusion, our survey demonstrates a high level of compliance with existing guidelines for monitoring and treatment of CMV and EBV in autologous HCT recipients for ADs. Nevertheless, PCR methods for monitoring and, more importantly, cut-off values for initiating pre-emptive therapy, may benefit from harmonisation in the future. The risk of CMV and EBV will vary by the underlying disease and by regimen intensity (including dose intensity of ATG), which needs to be evaluated in future multicentre studies.

## Supplementary information

Table S1

Participating centres

Survey details
