# Supplementary material for: Monitoring and management of CMV and EBV after autologous haematopoietic stem cell transplantation for autoimmune diseases: a survey of the EBMT Autoimmune Diseases Working party (ADWP)
Source: Bone Marrow Transplant. 2024 Nov 7;60(1):110–3. doi: 10.1038/s41409-024-02461-6 (PMC11726455; doi:10.1038/s41409-024-02461-6)
Supplement: Supplementary file 1 — Table S1 [file 41409_2024_2461_MOESM1_ESM.docx]

**Table S1**. Summary of continents and countries of responding centres. Shown are the number of participating centres per country and the percentage of responding centres per country/total centres.

| **Continent** | **Country** | **Number of centres** | **%** |
| --- | --- | --- | --- |
| America | Columbia | 1 | 1.82 |
| Asia | Israel | 1 | 1.82 |
|  | Singapore | 1 | 1.82 |
| Europe | Belgium | 2 | 3.64 |
|  | Czech Republic | 3 | 5.45 |
|  | Denmark | 1 | 1.82 |
|  | Finland | 1 | 1.82 |
|  | France | 6 | 10.91 |
|  | Germany | 7 | 12.73 |
|  | Greece | 1 | 1.82 |
|  | Italy | 11 | 20.00 |
|  | Lithuania | 1 | 1.82 |
|  | Russia | 2 | 3.64 |
|  | Spain | 3 | 5.45 |
|  | Sweden | 3 | 5.45 |
|  | Switzerland | 2 | 3.64 |
|  | The Netherlands | 2 | 3.64 |
|  | United Kingdom | 6 | 10.91 |
| Oceania | Australia | 1 | 1.82 |
