# Supplementary material for: Monitoring and management of CMV and EBV after autologous haematopoietic stem cell transplantation for autoimmune diseases: a survey of the EBMT Autoimmune Diseases Working party (ADWP)
Source: Bone Marrow Transplant. 2024 Nov 7;60(1):110–3. doi: 10.1038/s41409-024-02461-6 (PMC11726455; doi:10.1038/s41409-024-02461-6)
Supplement: Supplementary file 2 — Participating centres [file 41409_2024_2461_MOESM2_ESM.pdf]

We are grateful to all the EBMT affiliated centers contributing to this survey:

St. Vincent's Hospital, Kinghorn Cancer Centre, Sydney, Australia;
Antwerp University Hospital (UZA), Antwerp Edegem, Belgium;
University Hospital Gasthuisberg, Leuven, Belgium;
FOSCAL Internacional of the FOSUNAB Foundation, Floridablanca, Colombia;
University Hospital Ostrava, Ostrava, Czech Republic;
Charles University Hospital, Pilsen, Czech Republic;
Charles University Hospital, Prague, Czech Republic;
Rigshospitalet, Copenhagen, Denmark;
Kuopio University Hospital, Kuopio, Finland;
CHU Lapeyronie, Montpellier, France;
Hôpital Saint-Louis, APHP, Université Paris, Paris, France;
Hopital Saint Antoine, Paris, France;
Hopital La Miletrie, Poitiers, France;
ICANS - Institut de cancérologie Strasbourg Europe, Strasbourg, France;
CHU - Institut Universitaire du Cancer Toulouse, Toulouse, France;
Medizinische Klinik m. S. Hämatologie , Onkologie und Tumorimmunologie, Berlin, Germany;
ZSIS Universitaetsklinikum Knappschaftskrankenhaus Bochum GmbH, Bochum, Germany;
University of Freiburg, Freiburg, Germany;
University of Heidelberg, Heidelberg, Germany;
Universitaet Tuebingen, Tuebingen, Germany;
Universitaetsklinikum Wuerzburg, Wuerzburg, Germany;
George Papanicolaou General Hospital, Thessaloniki, Greece;
Rambam Medical Center, Haifa, Israel;
Ospedale Policlinico, Catania, Italy; Azienda Ospedaliera Universitaria Careggi, Firenze, Italy;
IRCCS Ospedale Policlinico San Martino, Genova, Italy;
Fondazione IRCCS - Ospedale Ospedale Maggiore Policlinico., Milano, Italy;
Fondazione IRCCS - Ospedale San Raffaele, Milano, Italy;
San Matteo Pavia Transplant Programme, Pavia, Italy;
Azienda Ospedaliero Universitaria Pisana, Pisa, Italy;
Ospedale Civile, Pescara, Italy;
Unità Operativa di Ematologia, Ravenna, Italy;

Grande Ospedale Metropolitano Bianchi Melacrino Morelli - Centro Unico Trapianti A. Neri, Reggio Calabria, Italy;

Ospedale Infantile Regina Margherita, Torino, Italy;

Vilnius University Hospital Santaros Klinikos, Vilnius, Lithuania;

Leiden University Hospital, Leiden, The Netherlands;

University Medical Centre, Utrecht, The Netherlands;

The Russian Federationn Children´s Research Hospital, Moscow, Russian Federation;

RM Gorbacheva Research Institute, Pavlov University, St Petersburg, Russian Federation;

Singapore General Hospital, Singapore, Singapore;

Hospital Clinic, Barcelona, Spain;

Hospital Clínico de Valencia, Valencia, Spain;

University Hospital La Fe, Valencia, Spain;

Skanes University Hospital, Lund, Sweden;

Karolinska University Hospital, Stockholm, Sweden;

University Hospital | Uppsala, Uppsala, Sweden;

University Hospital | Basel, Basel, Switzerland;

University Hospital, Zürich, Switzerland;

Imperial College Hammersmith London, London, United Kingdom;

University College London Hospital, London, United Kingdom;

London Bridge Hospital, London, United Kingdom;

Kings College Hospital London, London, United Kingdom;

Nottingham City Hospital, Nottingham, United Kingdom;

Sheffield Royal Hallamshire, Sheffield, United Kingdom.
