# Supplementary material for: Monitoring and management of CMV and EBV after autologous haematopoietic stem cell transplantation for autoimmune diseases: a survey of the EBMT Autoimmune Diseases Working party (ADWP)
Source: Bone Marrow Transplant. 2024 Nov 7;60(1):110–3. doi: 10.1038/s41409-024-02461-6 (PMC11726455; doi:10.1038/s41409-024-02461-6)
Supplement: Supplementary file 3 — Survey details [file 41409_2024_2461_MOESM3_ESM.pdf]

CMV and EBV monitoring policy of your centre after autologous HSCT for autoimmune diseases

Welcome to the EBMT survey on CMV and EBV monitoring policy of your centre for patients treated with autologous HSCT for autoimmune diseases.

Please complete the following questions regarding autoimmune disease patients currently managed in your centre.

The survey is divided into **4 different sections** and it includes **37 questions max** on **CMV and EBV monitoring and strategies of your centre for patients treated with autologous HSCT for autoimmune diseases.**

A. Contact information
B. CMV/EBV monitoring protocols
C. CMV
D. EBV

We would ask you to note that for some questions, where noted, it is possible to provide multiple responses.

All reports will be handled with strict anonymity. The information about the centre and the reporting person will only be known to the Paris Study Unit and will not be passed to the investigators.

Thank you for your participation - your involvement is **greatly appreciated !**

A. Contact information

1.Hospital name - Institution
2.City
3.Country
4.CIC - Center Identification Code
5.Contact person
6.Email adress
7.JACIE or FACT accreditation ☐ Yes ☐ No

B- CMV / EBV monitoring protocols

8. Do you perform autologous HSCT for autoimmune diseases in adult and/or pediatric patients in your centre?

☐ Only adult ☐ Only pediatric ☐ Both

9. Do you use different protocols for monitoring CMV in case of autologous HSCT for autoimmune diseases compared to hematologic indications?

☐ Yes ☐ No

10.Yes, please specify the main differences

……………………

11. Do you use different protocols for monitoring EBV in case of autologous HSCT for autoimmune diseases compared to hematologic indications?

☐ Yes ☐ No

12. Yes, please specify the main differences

…………………….

13. Do you use different protocols for monitoring CMV in case of different conditioning intensities used for HSCT in autoimmune diseases?

☐ Yes ☐ No

14. Yes, please specify the main differences

…………………………..

15. Do you use different protocols for monitoring EBV in case of different conditioning intensities used for HSCT in autoimmune diseases?

☐ Yes ☐ No

16. Yes, please specify the main differences

…………………………..

C- CMV

17. Do you monitor CMV in all HSCT recipients for AD?

☐ Only in autologous HSCT
☐ Only in allogeneic HSCT
☐ Both in autologous and allogeneic HSCT
☐ No

18. Do you monitor CMV-specific serology before autologous HSCT for AD?

☐ Yes ☐ No

19. Is CMV-viremia monitored by?

☐ Plasma
☐ Whole blood
☐ Other (Please specify ………….)

20. At which timepoints is CMV viremia monitored? (multiple choices allowed)

☐ At screening/pre-transplant
☐ At early follow up (within 100 days from HSCT)
☐ At late follow up (more than 100 days and within 180 days from HSCT)

☐ At long-term follow up (more than 180 days and within 1 year from HSCT)
☐ Other (Please specify ………)

21. How do you perform CMV surveillance in the early follow-up (within 100 days from HSCT)? *(multiple choices allowed)*
☐ PCR weekly in the whole period
☐ PCR twice a week in high-risk HSCT recipients (ie, patients with CMV DNAemia positivity and/or receiving additional immunosuppressive therapy for any cause)
☐ PCR weekly only in the first month after HSCT, then every other week in the second and third months
☐ Other

22. Is late and/or long-term CMV surveillance (more than 100 days after HSCT) performed?
☐ In all autologous HSCT AD recipients (i.e. every month)
☐ Only in presence of risk factors for late CMV disease

23. Do you have a threshold- cut off to start pre-emptive treatments for CMV reactivation?
☐ Yes ☐ No

24. If yes, specify threshold value and units.
…………………………..

25. Which antiviral prophylaxis recommended after autologous HSCT in AD recipient in your centre?
☐ Acyclovir
☐ Valacyclovir
☐ Other (Please specify ………….)

26.What are the strategies used for the treatment of CMV reactivation/disease in your centre? *(multiple choices allowed)*
☐ Ganciclovir
☐ Valganciclovir
☐ Foscarnet
☐ Cidofovir
☐ Letermovir
☐ Maribavir
☐ CMV-specific immunoglobulin
☐ Other (Please specify)

D- EBV

27.Do you monitor EBV in all HSCT recipients for AD?
☐ Only in autologous HSCT
☐ Only in allogeneic HSCT
☐ Both in autologous and allogeneic HSCT
☐ No

28. Do you monitor EBV-specific serology before autologous HSCT for AD?
☐ Yes ☐ No

29. Is EBV-viremia monitored by using
☐ Plasma

☐ Whole blood
☐ Other (Please specify ....)

30. At which timepoints is EBV viremia monitored? (multiple choices allowed)
☐ At screening/pre-transplant
☐ At early follow up (within 100 days from HSCT)
☐ At late follow up (more than 100 days and within 180 days from HSCT)
☐ At long-term follow up (more than 180 days and within 1 year from HSCT)
☐ Other (Please specify ………….)

31. How do you perform EBV surveillance in the early follow-up (within 100 days from HSCT)? *(multiple choices allowed)*
☐ PCR weekly in the whole period
☐ PCR twice a week in the whole period
☐ PCR twice a week only in high-risk HSCT recipients (ie, patients with EBV DNAemia positivity)
☐ PCR every other week in the whole period (every 2 weeks)
☐ PCR weekly only in the first month after HSCT, then every other week in the second and third months
☐ Other (Please specify …………)

32. Is late and/or long-term EBV surveillance (more than 100 days after HSCT) is performed
☐ In all autologous HSCT AD recipients (i.e. every month)
☐ Only in presence of risk factors for late EBV disease

33. Do you have a threshold- cut off to start pre-emptive treatments for EBV reactivation?
☐ Yes ☐ No

34. If yes, specify threshold value and units.
…………………………..

35. What are the strategies used for the treatment of EBV reactivation/disease in your centre? *(multiple choices allowed)*
☐ Reduction of concomitant immunosuppression (i.e. steroids)
☐ Rituximab
☐ High dose immunoglobulins
☐ Other (Please specify …………)

36. Do you monitor virus-specific T cells after autologous HSCT in AD recipient?
☐ Yes ☐ No

37. If yes, for which one? *(multiple choices allowed)*
☐ For CMV
☐ For EBV

Thank you for completing this survey.
